# CTLA-4 silencing in dendritic cells loaded with colorectal cancer cell lysate improves autologous T cell responses *in vitro*

**DOI:** 10.3389/fimmu.2022.931316

**Published:** 2022-08-01

**Authors:** Farid Ghorbaninezhad, Javad Masoumi, Mohammad Bakhshivand, Amir Baghbanzadeh, Ahad Mokhtarzadeh, Tohid Kazemi, Leili Aghebati-Maleki, Siamak Sandoghchian Shotorbani, Mahdi Jafarlou, Oronzo Brunetti, Mariacarmela Santarpia, Behzad Baradaran, Nicola Silvestris

**Affiliations:** ^1^ Immunology Research Center, Tabriz University of Medical Sciences, Tabriz, Iran; ^2^ Department of Immunology, Faculty of Medicine, Tabriz University of Medical Sciences, Tabriz, Iran; ^3^ Student Research Committee, Tabriz University of Medical Sciences, Tabriz, Iran; ^4^ Pharmaceutical Analysis Research Center, Tabriz University of Medical Sciences, Tabriz, Iran; ^5^ Medical Oncology Unit, Istituto di Ricovero e Cura a Carattere Scientifico (IRCCS) Istituto Tumori “Giovanni Paolo II” of Bari, Bari, Italy; ^6^ Medical Oncology Unit, Department of Human Pathology “G. Barresi”, University of Messina, Messina, Italy

**Keywords:** dendritic cell, T lymphocyte, colorectal cancer, tumor cell lysate, CTLA-4, cancer immunotherapy

## Abstract

Dendritic cell (DC)-based immunotherapy has increased interest among anti-cancer immunotherapies. Nevertheless, the immunosuppressive mechanisms in the tumor milieu, e.g., inhibitory immune checkpoint molecules, have been implicated in diminishing the efficacy of DC-mediated anti-tumoral immune responses. Therefore, the main challenge is to overcome inhibitory immune checkpoint molecules and provoke efficient T-cell responses to antigens specifically expressed by cancerous cells. Among the inhibitory immune checkpoints, cytotoxic T-lymphocyte-associated protein 4 (CTLA-4) expression on DCs diminishes their maturation and antigen presentation capability. Accordingly, we hypothesized that the expression of CTLA-4 on DCs inhibits the T cell-mediated anti-tumoral responses generated following the presentation of tumor antigens by DCs to T lymphocytes. In this study, we loaded colorectal cancer (CRC) cell lysate on DCs and inhibited the expression of CTLA-4 by small interfering RNA (siRNA) in them to investigate the DCs’ functional and phenotypical features, and T-cell mediated responses following DC/T cell co-culture. Our results demonstrated that blockade of CTLA-4 could promote stimulatory properties of DCs. In addition, CTLA-4 silenced CRC cell lysate-loaded DCs compared to the DCs without CTLA-4 silencing resulted in augmented T cell proliferation and cytokine production, i.e., IFN-γ and IL-4. Taken together, our findings suggest CTLA-4 silenced CRC cell lysate-loaded DCs as a promising therapeutic approach however further studies are needed before this strategy can be used in clinical practice.

## Introduction

Immunotherapy is a new alternative option for cancer treatment that has been developed due to advances in understanding various cancers pathogenesis (1). Unlike conventional therapies, immunotherapy manipulates and utilizes the patient’s own immune cells to fight cancer (2). Nowadays, cancer immunotherapies focus on specializing immune responses against tumors by involving dendritic cells (DCs) and stimulating anti-tumoral T-cell responses (3, 4). DCs are the immune system’s specialized antigen-presenting cells (APCs), important for linking the gap between innate and adaptive immunity, including the stimulation of anti-tumoral T cells (5, 6). The tumor-associated antigens (TAAs) which are processed and presented by DCs can activate anti-tumoral specific T cell responses (7). Various studies have indicated that DCs pulsed with a tumor cell lysate could provoke tumor antigen-specific T cell responses (8, 9). The induction of T cell-mediated anti-tumoral immunity through DCs reduces tumor volume and increases immunological memory to prevent cancer recurrence (3, 4).

As a result of DCs’ capability to initiate cellular immunity, they are promising candidates for cancer immunotherapy (10). Efficient DC-based cancer immunotherapy depends on the capacity of DCs to present TAAs to T cells, while its ineffectiveness is mostly related to the inhibitory immune checkpoint molecules, which make DCs incompetent (11). Among the inhibitory immune checkpoints expressed by DCs is cytotoxic T-lymphocyte-associated protein-4 (CTLA-4) (12). CTLA-4, which comprises three domains (ligand-binding region, transmembrane region, and cytoplasmic region) and a leading peptide, is an inhibitory molecule that can be expressed on various immune cells and modulate their function (13). It’s expression on DCs reduces their maturation and antigen presentation capacity (14). Furthermore, it has been reported that CTLA-4 stimulates the expression of inhibitory molecules, e.g., IL-10 and indoleamine 2,3-dioxygenase 1 (IDO1) in DCs (15).

Colorectal cancer (CRC) is the third most frequent cancer globally, accounting for approximately 935,000 deaths per year, and has been ranked as the second major cause of cancer deaths in 2020 (16). Chemotherapy, surgery, and radiotherapy are among the conventional treatments for this malignancy (17, 18). These conventional therapies may be related to adverse side effects, i.e., chemotherapy resistance, systemic toxicity, and cancer recurrence (2, 19). Most patients with CRC are constituted with Proficient Mismatch Repair (pMMR) and microsatellite stable (MSS) subtypes which have shown resistance to various therapies. The main reason for this is supposed to be antigen presentation weakness, diminished tumor-specific antigen expression, activation of immunosuppressive pathways, immune checkpoint signaling pathways, and presence of immune regulatory cells (20). Currently, various clinical trials in phases I/II or also III have been evaluating CTLA-4 targeted antibodies, including Ipilimumab and Tremelimumab, in different CRC subtypes, some of which have reported promising primary results. Phase II study of Ipilimumab and Nivolumab in combination with radiotherapy in MSS metastatic CRC has revealed that a combination of immune checkpoint inhibitors with radiation could significantly increase the disease control rate (21). First-line Nivolumab along with low-dose Ipilimumab, has demonstrated strong and durable clinical benefit and was well tolerated in Microsatellite Instability-High/Mismatch Repair-Deficient (MSI-H/dMMR) metastatic CRC patients in phase II study (22). Even though the Phase II study of the Tremelimumab in patients with refractory metastatic CRC had not demonstrated clinically meaningful effects (23), other studies have shown the improvement in its combination with other immune checkpoint inhibitors. In a randomized phase II study on refractory MSS CRC patients, the combination of Durvalumab and Tremelimumab has prolonged median overall survival by 2.5 months compared with patients who had received the best supportive care (24). The results of Phase II single-arm study of Durvalumab and Tremelimumab with concurrent radiotherapy showed an increase in circulating CD8^+^ T lymphocyte activation, differentiation, and proliferation in patients with pMMR metastatic CRC (25). It is worth to state that in 2018, the Food and Drug Administration (based on clinical trial NCT02060188) had approved ipilimumab for use in combination with nivolumab for the treatment of patients 12 years of age and older with MSI-H/dMMR metastatic CRC that had not responded to chemotherapy regimens fluoropyrimidine, oxaliplatin, and irinotecan.

Considering above mentioned issues, it seems that suppressing CTLA-4 expression on DCs along with loading these cells with a tumor cell lysate increases anti-tumoral specific T cell responses more efficiently, suggesting that this could be a possible and applicable cancer immunotherapy strategy. In this study, we demonstrated that CTLA-4 silencing in CRC cell lysate-loaded DCs enhances their stimulation and leads to boosted autologous T cells’ activation and cytokine production. Overall, these findings illustrate that CTLA-4-silenced tumor lysate-loaded DCs are a very attractive option for upgrading the effectiveness of DC vaccines in cancer immunotherapies.

## Materials and methods

### Materials

Complete media (CM) including RPMI 1640 (Gibco, USA, NY) that contains 10% Fetal Bovine Serum (FBS) (Gibco, USA, NY), Streptomycin 100 μg/mL, Penicillin 100 IU/mL (Gibco, USA, NY), 2 mmol/L of L-glutamine (Gibco, USA, NY). 2-mercaptoethanol (2ME) was ordered from Gibco (USA, NY). Recombinant human granulocyte macrophage colony stimulating factor (rh GM-CSF) was purchased from Sigma Chemical Co (Munich, Germany) and recombinant human interleukin-4 (rh IL-4) from eBioscience (CA, USA). Lipopolysaccharide (LPS) was ordered from Sigma Chemical Co (Munich, Germany). Carboxyfluorescein succinimidyl ester (CFSE) cell labeling kit was obtained from BioLegend (San Diego, United States). Human pan T cell isolation Kit was purchased from MiltenyiBiotec, Germany. Antibodies used to phenotype the cells were anti-HLA-DR-APC and anti-CD86- PerCP-cy5.5 from BioLegend (San Diego, United States), anti-CD40-CF-blue, anti-CD11c-FITC, and anti-CD14-FITC from Immunostep (Salamanca, Spain). Ficoll was obtained from Sigma Chemical Co (Munich, Germany). Bradford protein assay kit was purchased from Bio-Rad, (Hercules, CA).

### Tumor cell lysate preparation

Human CRC cell lines, including HT-29, HCT-116, and SW-480 cells, were purchased from the National Cell Bank of Iran (Pasteur Institute, Tehran, Iran). These cell lines were grown in CM and maintained at 37°C under humidification and 5% CO2. When the confluency of cultured cells reached 70-80%, they were detached using Trypsin, washed twice in serum-free media, and resuspended in sterile Phosphate Buffered Saline solution (PBS) at a concentration of 1×10^7^ cells/mL. Six rapid freeze-thaw cycles in liquid nitrogen and 37°C water bath were used to generate tumor cell lysates from cell suspensions. The produced lysate was then sonicated for 15 seconds to maximize the release of tumor antigens from lysed malignant cells. To remove cellular debris, the tumor cell lysates were centrifuged at 1500 rpm for 15 minutes at 4°C. The collected supernatant was passed through a 0.2-μm filter. The protein content in the lysates was determined using the Bradford assay. All lysates were maintained at -80°C until they were utilized.

### Peripheral mononuclear cells (PBMCs) isolation and DC generation

Fresh peripheral blood (PB) from three healthy individuals was collected in sterile falcons containing heparin, and PBMCs were isolated from these samples by fractionation over Ficoll gradients. The plastic adherence method was used to isolate monocytes from PBMCs. For this purpose, PBMCs were cultured at a concentration of 5×10^6^ per mL of serum-free RPMI-1640 medium in 6-well plates. Following 2 hours of incubation at 37°C, the non-adherent cells were washed off, and the adherent cells were cultured within the CM supplemented with 50 µM 2ME, 40 ng/mL, and 20 ng/mL of rh GM-CSF and rh IL-4, respectively. On days 2 and 4, the cultures were fed by removing half of the medium and replacing it with fresh CM containing rh GM-CSF and rh IL-4. After collecting immature DCs (iDCs) on day 6, 80 ng/mL of mixed human CRC cell lines lysate was added to the culture medium. After 5 hours of incubation at 37°C, 100 ng/mL of LPS was added to the culture medium. Tumor cell lysate-loaded mature DCs (mDCs) were generated after 24 hours of incubation at 37°C.

### Morphological and phenotypical characterization of DCs

The morphology of monocytes and DCs were observed, and photos were taken using an inverted light microscope (Optika, XDS-3, Italy). To analyze the phenotype of iDCs, mDCs, and CTLA-4-silenced mDCs, these cells were stained with specific surface markers including HLA-DR (anti-HLA-DR-APC), CD40 (anti-CD40-CF-blue), CD86 (anti-CD86- PerCP-cy5.5), and CD11c (anti-CD11c-FITC). The MACSQuant cytometer (Miltenyi Biotec, Auburn, CA, USA) was used to evaluate the cells, and the obtained data were analyzed using FlowJo software v10.5.3.

### siRNA preparation and transfection into DCs

CTLA-4-siRNA and transfection reagent were ordered from Santa Cruz Biotechnology (Santa Cruz, Canada). The sequence of ordered CTLA-4-siRNA is shown in **Table 1**. To obtain the optimum pulse voltage for CTLA-4-siRNA transfection, mDCs were harvested and subsequently transfected with different pulse voltages (160, 180, and 200 V) using Gene Pulser Xcell (Bio-Rad, USA). The transfection efficiency of siRNA in different pulse voltages was evaluated with FITC-labeled control siRNA (Santa Cruz Biotechnology, Santa Cruz, Canada). After obtaining a 160 V as the optimum pulse voltage for siRNA transfection, mDCs were transfected with different concentrations of CTLA-4-siRNA (40, 60, and 80 ρmol). Immediately after electroporation, mDCs were transferred into a 6-well plate containing CM. The relative expression of CTLA-4 was evaluated after 48 and 72 hours of incubation using quantitative real-time PCR (qRT-PCR). The optimum dose of siRNA and pulse voltage were determined for further experiments based on the provided results.

**Table 1:** List of primer sequences and siRNA.

Gene		Sequences
**CTLA-4 siRNA**	Sense Antisense	GUAUCUGAGUUGACUUGACAGAACA
		UGUCUGUCAAGUCAACUCAGAUACCA
**CTLA-4**	F R	TCAGTCCTTGGATAGTGAGGTTC
		TCAGTCCTTGGATAGTGAGGTTC
**TNF-α**	F R	TTCTCCTTCCTGATCGTGGCA
		TAGAGAGAGGTCCCTGGGGAA
**IL-10**	F R	AGGAAGAGAAACCAGGGAGC
		GAATCCCTCCGAGACACTGG
**T-bet**	F R	TCTCCTCTCCTACCCAACCAG
		CATGCTGACTGCTCGAAACTCA
**FOXP3**	F R	CAGCCAGTCTATGCAAACC
		GTCTTGTGTCAGTTTGAGGGTC
**GATA3**	F R	GCATCCAGACCAGAAACCGAA
		TCGCGTTTAGGCTTCATGATACT
**18S**	F R	CTACGTCCCTGCCCTTTGTACA ACACTTCACCGGACCATTCAA

### Autologous CD3^+^ T cells isolation and CFSE labeling

The separation of autologous CD3^+^ T cells from PBMCs of the same individuals used for DC generation was performed by magnetic activated cell sorting (MACS) using a human Pan T Cell Isolation Kit according to the manufacturer’s instructions. Briefly, following isolating the PBMCs, the cell suspension was centrifuged for 10 minutes at 300 g. The supernatant was removed, and then 40 μl MACS buffer and 10 μL of pan T cell biotin Ab cocktail were added per 1×10^7^ total cells. After incubating for 5 min at 2−8°C, 30 μL of MACS buffer and 20 uL of Pan T Cell Microbead cocktail were added per 1×10^7^ total cells. Following a 10-minute incubation at 2−8°C, cells were washed with 1−2 mL of MACS buffer and resuspended in 500 uL of MACS buffer. After placing the MACS column in the MACS separator’s magnetic field, the cell suspension was added to this column. Negatively selected CD3^+^ T cells were unlabeled cells that had passed through the column. CFSE labeling of isolated CD3^+^ T cells was performed according to the protocol provided by the manufacturer. In brief, purified T cells were resuspended in PBS and incubated with CFSE at a concentration of 5 μM for 5 minutes at room temperature in the dark. The reaction was quenched by adding RPMI-1640 medium containing 20% FBS. After the final washing step, the cells were resuspended in pre-warmed cell culture media.

### CD3^+^ T-cells’ proliferation assay

To assess mDCs and CTLA-4-silenced-mDCs for their ability to stimulate the proliferation of autologous T cells, DC-T cell co-culture was performed. mDCs and CTLA-4-silenced-mDCs as stimulator and CFSE-labeled autologous CD3^+^ T cells as responder cells were co-cultured in the ratios of 1:5 and1:10 in V bottom 96-well plate. T cells activated by phytohemagglutinin (PHA) (5%) (Sigma Chemical Co., Munich, Germany) were served as a positive control, whereas co-cultured iDCs with T-cells were considered as an unstimulated group. After 4 days of incubation at dark conditions, flow cytometry was used to analyze the proliferation of the CFSE-labeled T cells. Unlabeled CD3^+^ T cells were used as unstained.

### Cytokine assay

To evaluate the capability of mDCs and CTLA-4-silenced-mDCs to promote cytokine production in autologous T cells, freshly isolated CD3^+^ T cells were cultured with mDCs and CTLA-4-silenced-mDCs in the ratios of 1:5 in 24-well plate. The supernatants of the co-cultures were obtained 48 hours after stimulation with DCs, and the quantities of IFN-γ, IL-4, and TGF-β were measured using commercial ELISA kits (R&D Systems, Minnneapolis, MN, USA). As well, IL-12 and IL-10 levels were evaluated in supernatants of the mDCs and CTLA-4-silenced-mDCs cultures using ELISA kits (R&D Systems, Minnneapolis, MN, USA).

### RNA isolation and qRT-PCR

Total cellular RNA was extracted using the TRIzol reagent (Roche Diagnostics, Mannheim, Germany) according to the manufacturer’s guidelines. The concentration of RNA was then measured by a spectrophotometer. The RNA was maintained at -80°C, and the Complementary DNA (cDNA) was synthesized using a BioFACT 2step 2X RT-PCR Pre-Mix (Taq), and the Applied Biosystems StepOnePlusTM Real-Time PCR System (Life Technologies, Carlsbad, CA, USA) was used to assess the expression of all genes in this manuscript. To normalize the expression of target mRNAs, the 18s gene was employed as an internal control. The sequences of primers are provided in **Table 1**. All reactions were carried out in triplicate, and the relative mRNA expression was calculated using the 2^-ΔΔCt^ method.

### Statistical analysis

All the raw data were analyzed with GraphPad Prism v8.0.2 (GraphPad Software, San Diego, California USA). Student’s t-test and One-way ANOVA test were used to compare data between two and more than two groups, respectively. Each parameter was measured in triplicate, and data of each group were expressed as mean ± SD with the significance cut-off of p-value ≤ 0.05 (ns: not significant; *: P≤ 0.05; **: P≤ 0.01; ***: P≤ 0.001; and ****: P≤ 0.0001).

## Results

### siRNA transfection in mDCs significantly decreased the gene expression of CTLA-4

**Figure 1:**
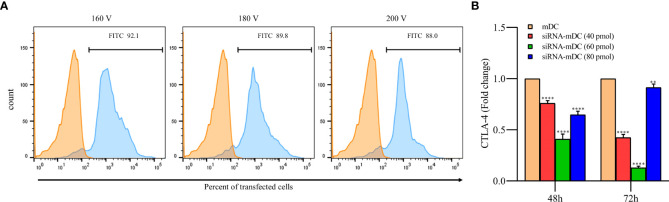
siRNA transfection outstandingly silenced CTLA-4 gene in mDCs. **(A)** Percentages of transfected mDCs obtained for the different pulse voltages. More than 92% of the mDCs were transfected with FITC-labeled control siRNA at 160 V. The viability of all transfected and un-transfected DCs, determined by the Trypan blue exclusion test, was above 90 percent. **(B)** CTLA-4 mRNA expression was suppressed in mDCs after 72 hours of transfection with 60 pmol of CTLA-4 siRNA compared with untransfected mDCs; (**P≤ 0.01 and ****P≤ 0.0001). CTLA-4, Cytotoxic T-lymphocyte-associated protein-4; mDCs, Tumor cell lysate-loaded mature dendritic cells; siRNA-mDCs, CTLA-4-silenced mDCs.

To achieve the optimum voltage for transfection, mDCs were transfected with different pulse voltages (160, 180, and 200 V). There was no significant difference in transfection rate between selected voltages, and it is shown to be near 90% in all of them. Therefore, the least voltage (160V) was selected in order to reduce stress in cells during pulsing (**Figure 1A**). Furthermore, after the transfection of CTLA-4-siRNA in different concentrations into mDCs, to assess siRNA effectiveness in gene silencing, the qRT-PCR was used. Compared with untransfected mDCs, which is considered as a control group, 60 pmol of CTLA-4 siRNA compared to 40 and 80 pmol more significantly reduced CTLA-4 mRNA expression in transfected cells at both 48 and 72 h incubation time (**Figure 1B**, P≤ 0.0001). As a result, the following experiments were conducted using a 60 pmol as the optimal dose of CTLA-4-siRNA and 160 V as the optimal transfection voltage.

### CTLA-4 silencing significantly increased maturation and activation of DCs

**Figure 2:**
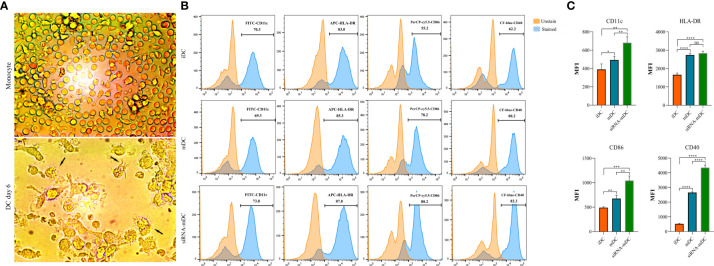
Morphological and phenotypical characterization of DCs. **(A)** Morphological changes during *in vitro* culture of adherent monocytes and differentiated DCs. Arrows indicate DCs with typical morphology having sharp dendrites. **(B)** Phenotypic characterization of iDCs, mDCs, and CTLA-4-silenced mDCs quantified by flow cytometry for the expression of surface markers, including CD11c, HLA-DR, CD86, and CD40. Results are expressed as the percentage of stained cells for these markers (figures **(A, B)** provided as representative of all samples). **(C)** The expression levels of CD11c, HLA-DR, CD86, and CD40 between iDCs, mDCs, and CTLA-4-silenced mDCs are represented as MFI. (ns, not significant, *P≤ 0.05, **P≤ 0.01, ***P≤ 0.001, and ****P≤ 0.0001). DCs, dendritic cells; CTLA-4, Cytotoxic T-lymphocyte-associated protein-4; iDCs, Immature dendritic cells; mDCs, Tumor cell lysate-loaded mature dendritic cells; siRNA-mDCs, CTLA-4-silenced mDCs; HLA-DR, Human leukocyte antigen-DR isotype; MFI, Median fluorescence intensity.

**Figure 3:**
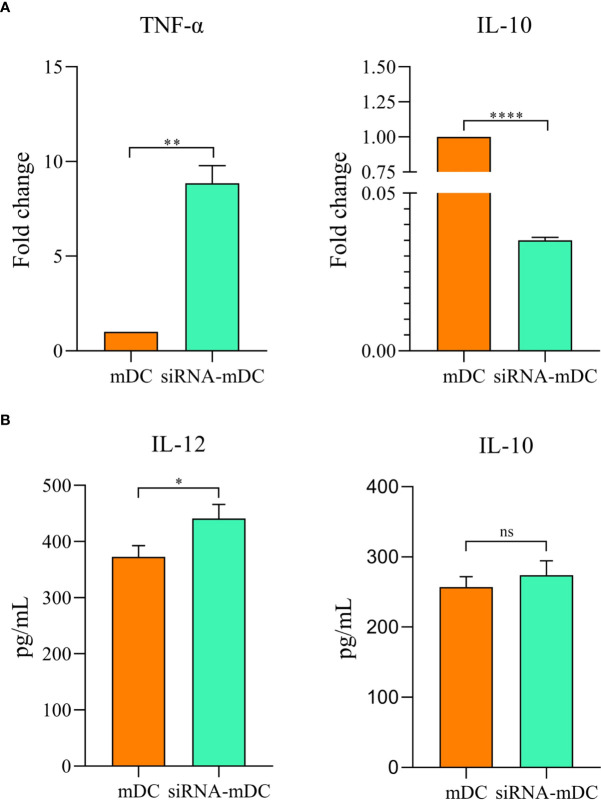
Cytokine expression and secretion profile in mDCs and CTLA-4-silenced-mDCs. The expression levels of TNF-α and IL-10 **(A)** were assessed by qRT-PCR. IL-12 and IL-10 **(B)** quantities in the cell culture supernatants was evaluated by ELISA; (ns, not significant, *P≤ 0.05, **P≤ 0.01, and ****P≤ 0.001). CTLA-4, Cytotoxic T-lymphocyte-associated protein-4; mDCs, Tumor cell lysate-loaded mature dendritic cells; siRNA-mDCs, CTLA-4-silenced mDCs; qRT-PCR, Quantitative Real-time polymerase chain reaction; ELISA, Enzyme-linked immunosorbent assay.

Microscopic analysis revealed morphological changes during *in vitro* culture of adherent monocytes and differentiated DCs (**Figure 2A**). Using the surface expression of markers related to DC maturation and antigen presentation, phenotypic evaluation of iDCs, mDCs, and CTLA-4-silenced mDCs was performed as detailed in the “Materials and methods” section. Flow cytometry analysis showed that all three of these cells had typical expressions of CD11c, HLA-DR, CD86, and CD40 (**Figure 2B**). We further analyzed the differences in the surface expression of these markers between these three groups of DCs based on median fluorescence intensity (MFI). Conversion of iDCs to mDCs increased the surface expression of CD11c (P≤ 0.05), HLA-DR (P≤ 0.0001), CD86 (P≤ 0.01), and CD40 (P≤ 0.0001) markers (**Figure 2C**). CTLA-4 suppression in mDCs, significantly elevated the expression of CD11c (P≤ 0.01), CD86 (P≤ 0.01), and CD40 (P≤ 0.0001) compared with mDCs, whereas increased HLA-DR expression was not significantly different (**Figure 2C**). Furthermore, activated DCs are known to produce inflammatory cytokines. Accordingly, to further characterize the impact of CTLA-4 silencing in the activation of mDCs, the concentration of IL-12 and IL-10 in the cell culture supernatants and the expression of TNF-α and IL-10 mRNAs were evaluated by ELISA and qRT-PCR, respectively. According to the findings, CTLA-4 silencing resulted in enhanced TNF-α (**Figure 3A**, P≤ 0.01) and decreased IL‐10 (**Figure 3A**, P≤ 0.0001) expression in mDCs. Also, compared with mDCs, IL-12 levels in the cell culture supernatants were increased after CTLA-4 inhibition (**Figure 3B**, P≤ 0.05). Interestingly, IL-10 was higher in the supernatants of CTLA-4-silenced mDCs compared with mDCs, but the difference was not significant (**Figure 3B**).

### Deletion of CTLA-4 in DCs improved T-cell responses

**Figure 4:**
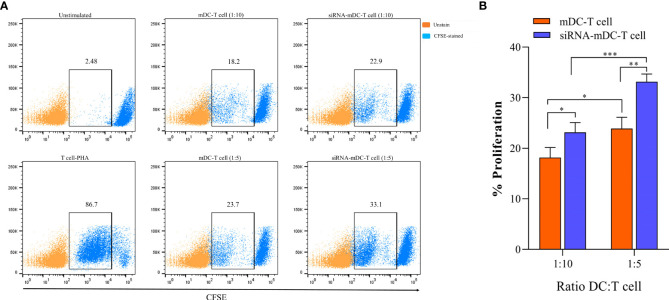
CTLA-4-silenced-mDCs significantly increase CD3+ T cells’ proliferation. **(A)** The percentage of CFSE-labeled autologous CD3^+^ T cells provoked by mDCs and CTLA-4-silenced-mDCs at a 1:5 and 1:10 DC/T cell ratio were determined by FACS *via* calculating the CFSE loss. **(B)** Enhanced mDC’s capacity in T cell proliferation following CTLA-4 knockdown; (*P≤ 0.05, **P≤ 0.01, and ***P≤ 0.001). CTLA-4, Cytotoxic T-lymphocyte-associated protein-4; mDCs, Tumor cell lysate-loaded mature dendritic cells; siRNA-mDCs, CTLA-4-silenced mDCs; FACS, Fluorescent activated cell sorting.

**Figure 5:**
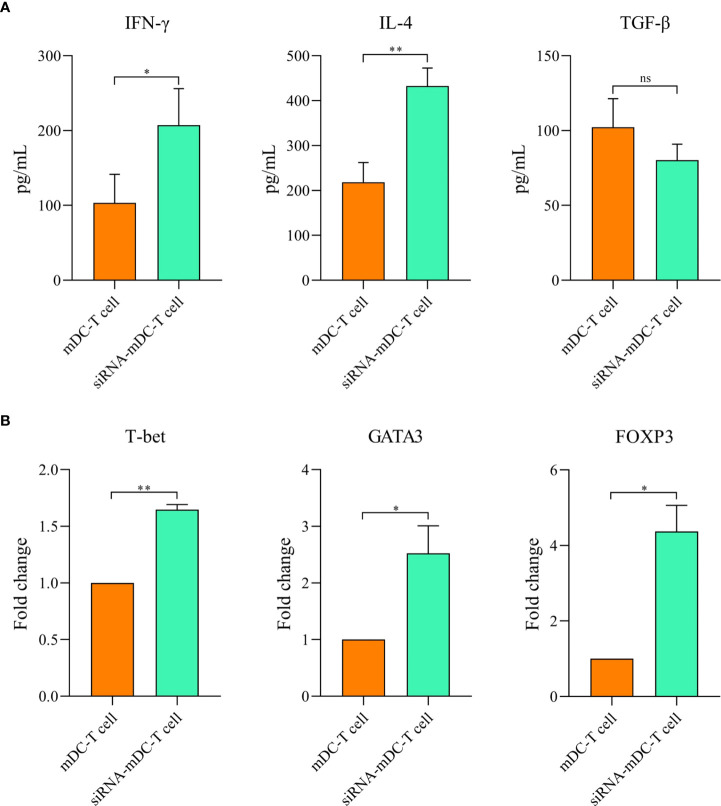
Silencing of CTLA-4 in mDCs strengthened T cell-mediated effector functions. Cytokine production by T cells following co-culture with mDCs and CTLA-4-silenced-mDCs. **(A)** The DC/T cell co-culture supernatants were evaluated for IFN-γ, IL-4, and TGF-β secretion by CD3^+^ T cells by ELISA. **(B)** Expression analysis of T cell-associated transcription factors, i.e., GATA3, T-bet, and FOXP3 by CD3^+^ T cells following co-culture with mDCs and CTLA-4-silenced-mDCs were determined *via* qRT-PCR; (ns, not significant, *P≤ 0.05, and **P≤ 0.01). CTLA-4, Cytotoxic T-lymphocyte-associated protein-4; DC, dendritic cell; mDCs, Tumor cell lysate-loaded mature dendritic cells; siRNA-mDCs, CTLA-4-silenced mDCs; ELISA, Enzyme-linked immunosorbent assay; GATA3, GATA binding protein 3; T-bet, T-box protein expressed in T cells; FOXP3, Forkhead box P3; qRT-PCR, Quantitative reverse transcription polymerase chain reaction.

To determine the impact of CTLA-4 suppression in mDCs, on the T cell anti-tumor activity, proliferation, and cytokine secretion of CD3^+^ T cells were assessed in the following experiments. Co-culture assay was done with mDCs and CTLA-4-silenced-mDCs as stimulator and CFSE-labeled autologous CD3^+^ T cells as a responder in 1:5 and 1:10 ratios to assess proliferation as previously described in the “Materials and methods” section. The results indicated that compared to 1:10, the ratio of 1:5 resulted in increased T cell proliferation in all groups (P≤ 0.05) (**Figures 4A, B**). Furthermore, in both 1:10 and 1:5 ratios, CTLA-4-silenced mDCs showed a higher capacity to stimulate CD3^+^ T cell proliferation than mDCs (P≤ 0.05 and P≤ 0.01, respectively) (**Figures 4A, B**). In addition, the anti-tumor activity of T cells was assessed by measuring IFN-γ, IL-4, and TGF-β levels in the supernatant of T cell/DC co-cultures. Co-cultures of autologous T cells and CTLA-4-silenced-mDCs resulted in considerably higher IFN-γ (**Figure 5A**, P≤ 0.05) and IL-4 (**Figure 5A**, P≤ 0.01) levels than T cell/mDCs co-culture, which is Coordinated with the increased proliferation of CD3^+^ T cells. TGF-β levels were diminished in the supernatants of T cell/CTLA-4-silenced mDCs co-culture compared with T cell/mDCs co-culture, but the difference was not significant (**Figure 5A**). The findings are consonant with enhanced GATA3 and T-bet mRNA expression in T cells purified from the T cell/CTLA-4-silenced-mDCs co-culture compared with T cell/mDCs co-culture (**Figure 5B**). Interestingly, FOXP3 mRNA was found to be considerably higher in T cells purified from the T cell/CTLA-4-silenced-mDCs co-culture than in T cell/mDCs co-culture (**Figure 5B**).

## Discussion

There is considerable interest in utilizing DCs to stimulate antigen-specific anti-tumoral CD4^+^ and CD8^+^ T cell responses in cancer immunotherapy. In this regard, synthetic peptides or proteins obtained from TAAs, i.e., MUC1, Her-2/neu, tyrosinase, CEA, and Melan-A/MART, can be loaded into DCs to stimulate antigen-specific T cells. Vaccinating against a single antigen has limitations since it’s unclear whether the defined antigens can trigger an effective anti-cancer immunity (9). Tumor cell lysates are a reliable source of tumor antigens, particularly for malignancies without tumor-specific antigens, which can be presented to T cells by DCs *via* the MHC class I and class II molecules (26). As a result, lysate-pulsed DCs are more potent to trigger T cell activation. Nevertheless, several immunosuppressive mechanisms utilized by cancerous cells in their milieu cause abnormalities in the function of DCs, which contribute to tumor cell escape from the immune system and diminish the efficacy of DC-based immunotherapy. Inhibitory immune checkpoint molecules are regarded as key participants in the TME’s immune‐modulatory scenario (27). Among these molecules, the importance of CTLA-4 in the inhibition of anti-tumor T cell responses has been subjected to the argument for over a decade. CTLA-4 is a T cell activation inhibitory receptor that binds to the B7 ligand family, CD80 and CD86, on the surface of APCs with a high affinity and disrupts CD28-mediated signaling to T cell (28). When CTLA-4 is inhibited, CD28 binds to CD80/CD86, enhancing CD4^+^ and CD8^+^ T cell-mediated immunity (29). According to various studies, CTLA-4 is expressed in non-T cells like DCs and has immunomodulatory effects on them (12, 14). CTLA-4 expression in DCs decreases their maturation by reducing CD83 expression. Furthermore, CTLA-4-expressing DCs have a reduced ability to present antigens to T cells (14). IL-10 upregulates following CTLA-4 engagement on DCs, while IL-8 and IL-12 production diminishes (12).

Several investigators have attempted to pulse DCs with tumor cell lysates (30, 31) or suppress inhibitory immune checkpoints, e.g., PD-L1/PD-L2 (32, 33) in DCs to increase the efficacy of DC-based cell therapy outcomes. However, none of them evaluated the effect of tumor antigen loading on DCs as well as CTLA-4 inhibition in them concomitantly. Since CTLA-4 is expressed on DCs and has a significant effect on diminishing their function, we aimed to inhibit the expression of CTLA-4 in CRC cell lysate-pulsed-DCs *via* transfection of siRNA to enhance the efficacy of DC-based cancer immunotherapy. We used CTLA-4 siRNA duplexes, which have longevity, stability, and remarkable specificity, to suppress CTLA-4 expression on CRC cell lysate-loaded monocyte-derived DCs.

Our results showed that in comparison to untransfected mDCs, CTLA-4 mRNA expression in transfected mDCs was significantly inhibited after 72 hours of transfection with 60 pmol of CTLA-4 siRNA at 160 V (**Figure 1B**). After determining the optimal dose and pulse voltage required for siRNA transfection into mDCs, we investigated the stimulatory impact of CTLA-4 suppression in mDCs on their antigen-presenting features by evaluation of surface molecule expression patterns and cytokine secretion characteristics. Hence, despite the non-significant increase in HLA-DR expression, our results indicated that inhibition of CTLA-4 in mDCs significantly amplified the expression of CD11c, CD40, and CD86 (**Figure 2C**), implying the potential of CTLA-4-silenced mDCs to stimulate T lymphocytes. Another important signal for T cell activation is cytokines released by DCs. Activated DCs produce inflammatory cytokines, i.e., TNF-α and IL-12, while the production of IL-10 is the hallmark of tolerogenic DCs (34). According to our results, following CTLA-4 knockdown in mDCs, the expression of TNF-α and IL-10 (**Figure 3A**) was increased and decreased, respectively. In addition, after CTLA-4 suppression in mDCs, the quantities of IL-12 in the cell culture supernatants were significantly amplified. In contrast, the increase in the quantities of IL-10 in the supernatants of CTLA-4-silenced mDCs was not significant (**Figure 3B**).

Since antigen-loaded DCs can stimulate T cell-mediated immunity, we further investigated the stimulatory capacity of our CTLA-4-silenced mDCs in antigen-specific T-cell responses. Based on our results, CRC cell lysate loading in DCs along with CTLA-4 inhibition significantly increased CD3^+^ T cell proliferation in autologous co-culture assay compared with DCs where only loaded with CRC cell lysate (**Figure 4B**). The effect of CTLA-4 inhibition in DCs on cytokine secretion profile by T cells was also determined. The results showed that suppression of CTLA-4 considerably increased the production of IFN-γ, a marker associated with T helper type 1, and IL-4 (**Figure 5A**), a marker associated with T helper type 2. However, the decrease in the TGF-β (marker of regulatory T cell) levels following T cell/CTLA-4-silenced mDCs co-culture was not significant (**Figure 5A**). Next, we evaluated the expression of transcription factors related to the T cell responses. Enhanced GATA3 (T helper 2 marker) and T-bet (T helper 1 marker) mRNA expression in CD3^+^ T cells co-cultured with CTLA-4-silenced-mDCs was observed (**Figure 5B**). Surprisingly, the expression of FOXP3 mRNA, a transcription factor related to regulatory T cells, was increased in T cell/CTLA-4-silenced-mDCs co-culture (**Figure 5B**).

**Figure 6:**
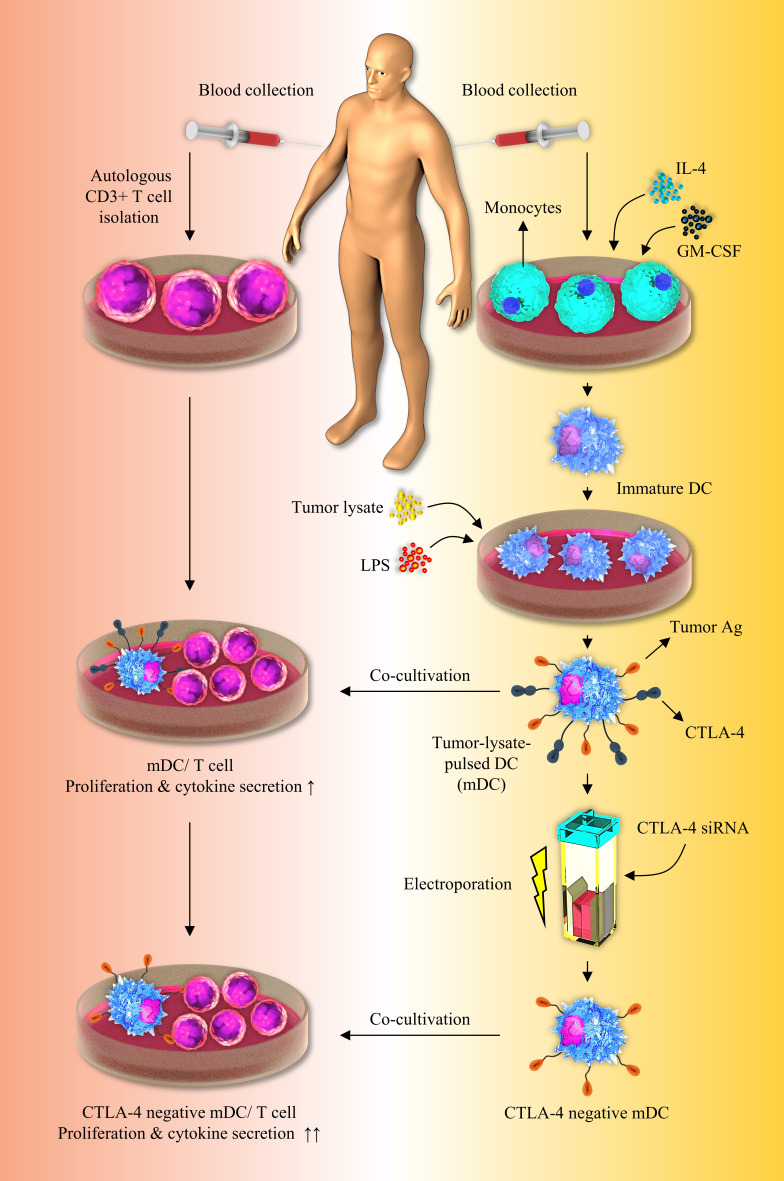
Inhibition of CTLA-4 molecules augments T-cell responses to tumor-lysate-pulsed-DCs. Silencing of CTLA-4 gene in DCs *via* using siRNA improves their stimulatory properties. Co-culture of tumor-lysate-pulsed CTLA-4-scilenced DCs with CD3^+^ T cells improves T-cell mediated responses. CTLA-4, Cytotoxic T-lymphocyte-associated protein-4; DC, dendritic cell; siRNA, small interfering RNA.

As previously stated, various investigators have attempted to separately load tumor cell lysates on DCs or silence inhibitory immune checkpoints, such as PD-L1/PD-L2, in order to develop effective DC-based cell therapy. Aerts et al. have demonstrated that following the use of tumor lysate–loaded DCs, the tumor-specific T-cell response was established by the production of IFN-γ in the mesothelioma-murine model, which was consistent with our findings (35). In accordance with our results, it was reported that T cells release higher quantities of IFN-γ and show a higher rate of proliferation when they are stimulated by apoptotic tumor cell-loaded DCs (8). In another study, it has been indicated that human gastric tumor lysates loaded DCs can boost the proliferation of CD3^+^ T cells (36). In accordance with our results, Schnurr et al. have reported the increased IL-12 secretion by panc-1 tumor cell lysates loaded DCs (37). In addition, in a breast tumor-bearing human-SCID model, suppression of PD-L1 boosted DC maturation, proliferation, and IL-12 secretion, as well as T-cell-mediated responses (38). Roeven and colleagues have reported that suppression of PD-L1/PD-L2 in human monocyte-derived DCs significantly boosted *ex vivo* antigen-specific T-cell responses (39). Furthermore, Van den Bergh et al. have indicated that PD-L1/2-silenced DCs exhibited an increased capacity to enhance T-cell proliferation and TNF-α production than normal DCs, which was consistent with our findings (32). Oh and colleagues have shown that PD-L1 elimination in DCs improves anti-tumor CD8^+^ T-cell responses (40). Some studies have reported that in the high stimulatory conditions like exposure to mature autologous DCs or stimulation with CD3, CD4^+^ T cells acquire regulatory properties including the FOXP3 expression, while producing effector cytokines like IFN-γ, IL-2, IL-4 and IL-10 (41–44). This may be the reason laying behind the FOXP3 high expression in T cell/CTLA-4-silenced-mDCs co-cultures. Although in previous studies, DCs were separately loaded with tumor lysates or suppressed for inhibitory immune checkpoints like PD-L1/PD-L2, however, there is no study regarding the concomitant silencing of CTLA-4 and tumor cell lysate loading on DCs in order to boost the effectiveness of DC-based immunotherapy. Our result showed that CTLA-4 knockdown in CRC cell lysate-loaded DCs enhances autologous T cell activation and cytokine secretion, implying a promising therapeutic option for future preclinical and clinical investigations (**Figure 6**).

## Conclusion

Despite DCs having been loaded with tumor lysates or inhibited for inhibitory immune checkpoints in previous studies, there has been no investigation on the simultaneous silencing of CTLA-4 and tumor cell lysate loading on DCs. This study has implied that CTLA-4 knockdown in CRC cell lysate-loaded DCs remarkably improves their maturation and stimulatory activity. Furthermore, these modified DCs can robustly enhance the activation and cytokine secretion of co-cultured T-cells more than DCs where only pulsed with tumor lysate. As a result of these findings, it is suggested that this anti-cancer therapeutic strategy be investigated further in preclinical investigations in order to confirm this concept.

## Data availability statement

The raw data supporting the conclusions of this article will be made available by the authors, without undue reservation.

## Ethics statement

The studies involving human participants were reviewed and approved by ethical code: IR.TBZMED.REC.1399.938. The patients/participants provided their written informed consent to participate in this study. Written informed consent was obtained from the individual(s) for the publication of any potentially identifiable images or data included in this article.

## Author contributions

FG is the first author of the manuscript, performed the majority of experiments, and wrote the initial version of the manuscript; JM contributed to cellular assays, data analysis, and revised the manuscript; MB contributed to data analysis and manuscript preparation; AB contributed to molecular assays; OB, AM, TK, LA-M, SS, and MS commented on the manuscript and provided critical feedback; BB and NS are the corresponding authors of the manuscript, supervised the project, and also contributed to the revising of the main text of the manuscript. All authors contributed to the article and approved the submitted version.

## Funding

This study was supported by the Immunology Research Center, Tabriz University of Medical Sciences, Tabriz, Iran (Grant numbers: 66133 & 60603).

## Conflict of interest

The authors declare that the research was conducted in the absence of any commercial or financial relationships that could be construed as a potential conflict of interest.

## Publisher’s note

All claims expressed in this article are solely those of the authors and do not necessarily represent those of their affiliated organizations, or those of the publisher, the editors and the reviewers. Any product that may be evaluated in this article, or claim that may be made by its manufacturer, is not guaranteed or endorsed by the publisher.
